# Supplementary material for: Clinicopathological Pearls and Diagnostic Pitfalls in IgG4-Related Disease: Challenging Case Series and Literature Review
Source: Diagnostics (Basel). 2025 Sep 10;15(18):2299. doi: 10.3390/diagnostics15182299 (PMC12469046; doi:10.3390/diagnostics15182299)
Supplement: Supplementary file 1 [file diagnostics-15-02299-s001.zip › diagnostics-3782755-supplementary.pdf]

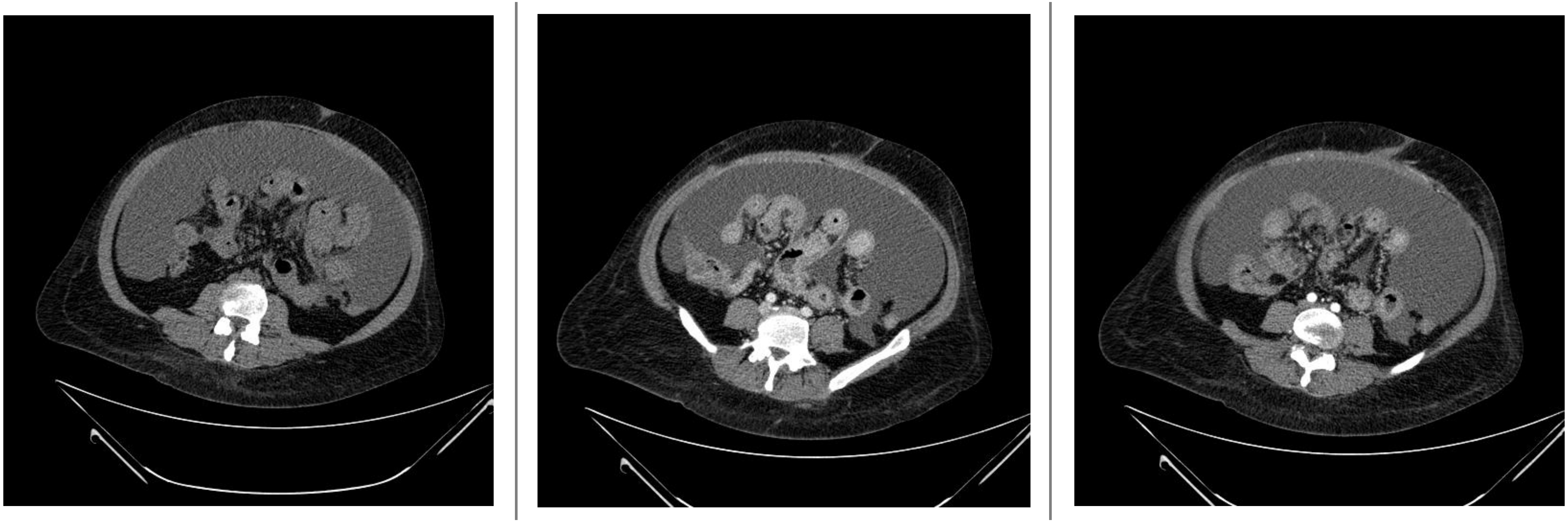

A B C

Figure S1, CT scan, section on bowel.

A: non contrast enhanced – B: contrast enhanced, venous phase – C: contrast enhanced, arterial phase

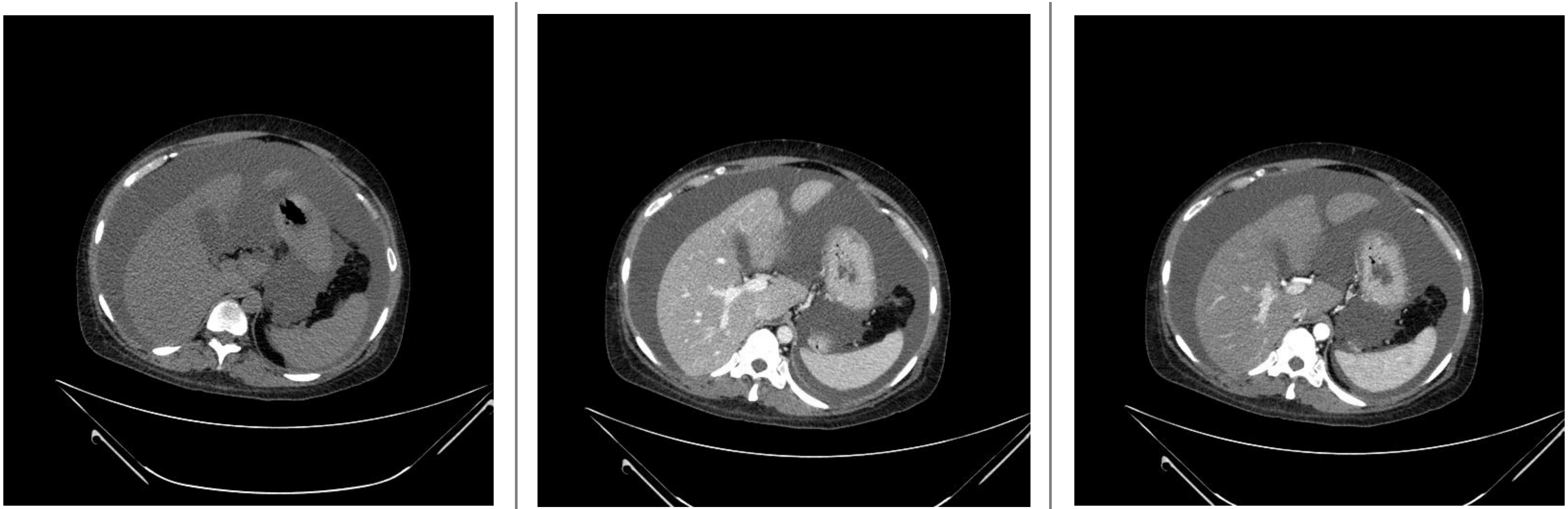

A B C

Figure S2, Gastro-enteric IgG4 related disease. Abdomen CT scan, section on stomach.
A: non contrast enhanced – B: contrast enhanced, venous phase – C: contrast enhanced, arterial phase

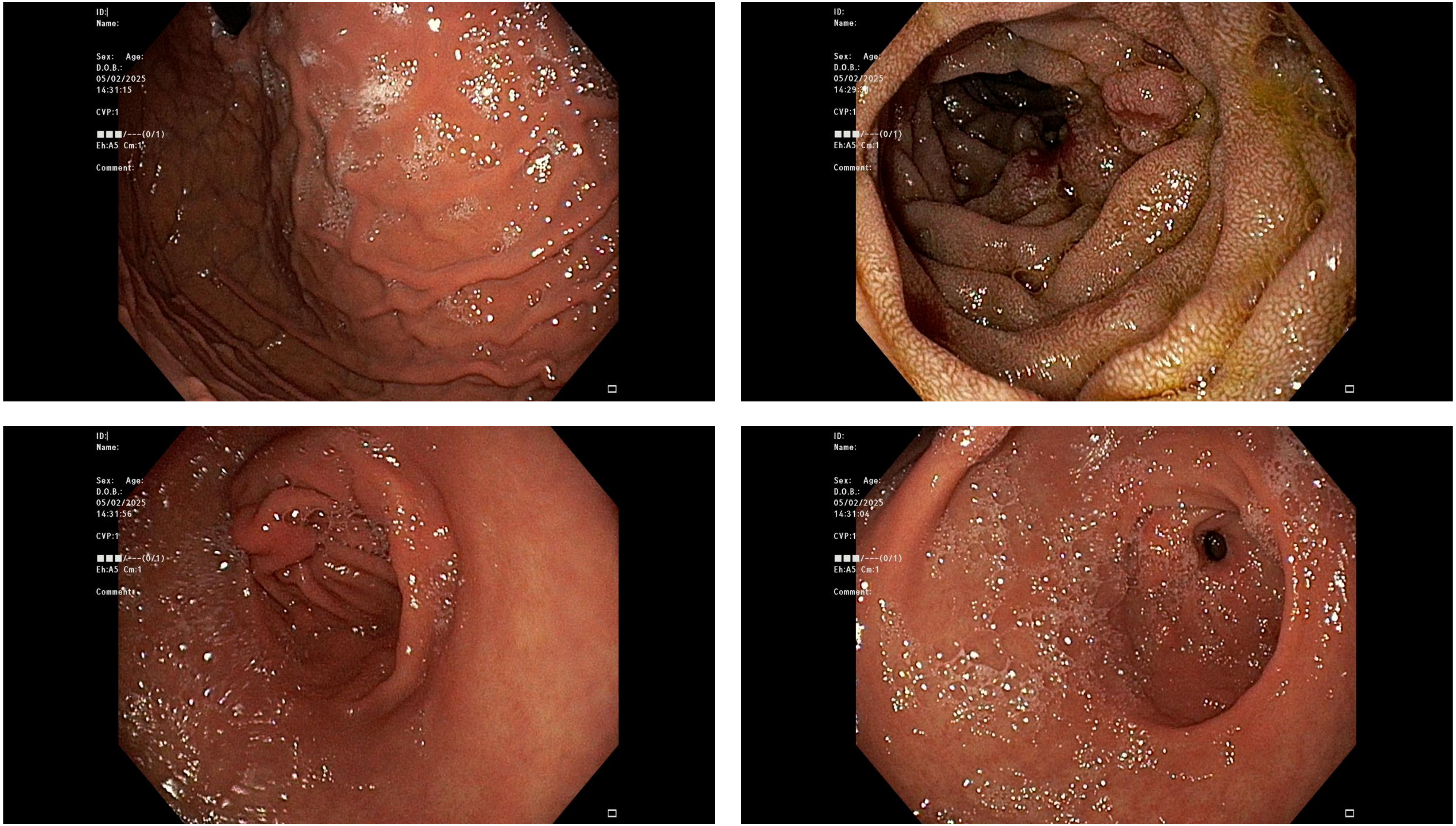

Figure S3, Gastroenteric IgG4 related disease. EGDS pictures.

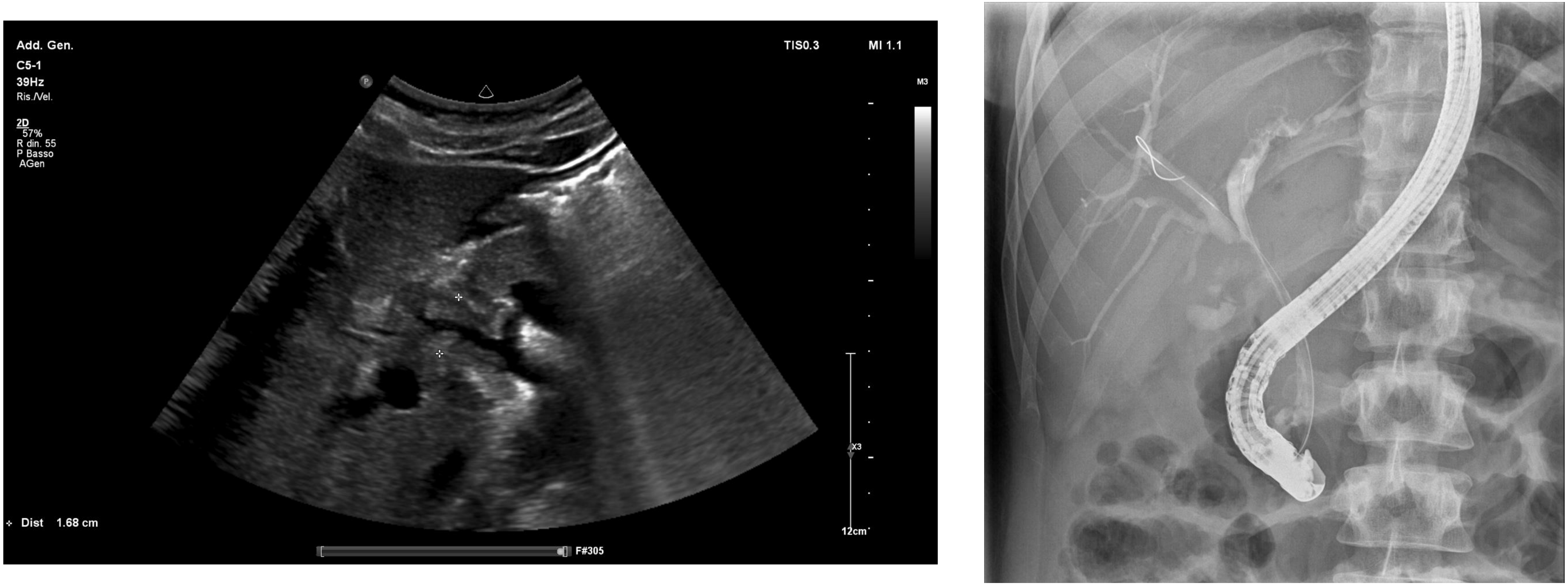

A

B

Figure S4, Sclerosing cholangitis IgG4 related disease.
A: Endoscopic Ultrasound - B: ERCP

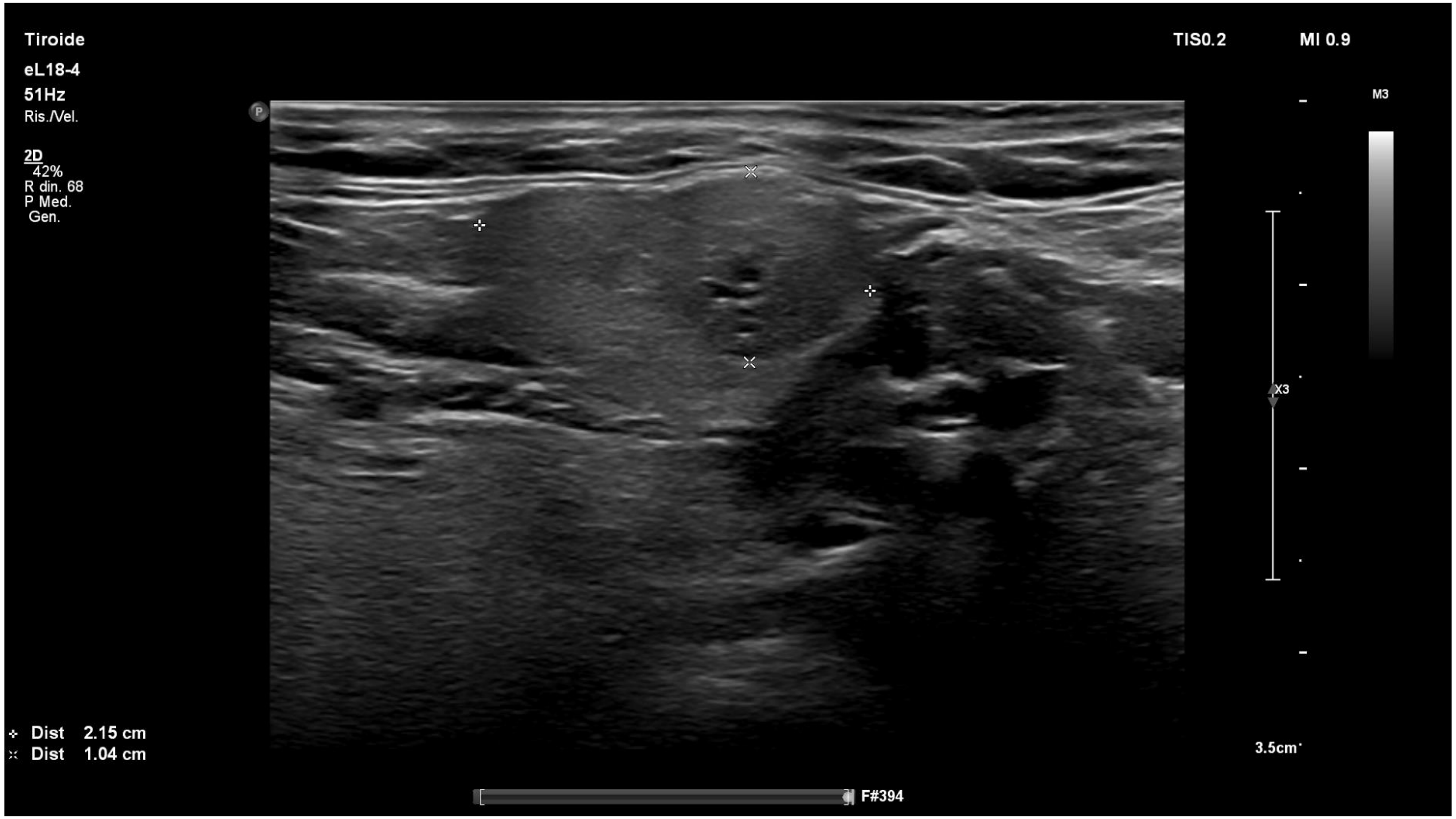

Figure S5, Salivary IgG4 related disease. Head-Neck ultrasonography
